# Shape Approximation and Size Difference of the Upper Part of the Talus: Implication for Implant Design of the Talar Component for Total Ankle Replacement

**DOI:** 10.1155/2022/1248990

**Published:** 2022-01-12

**Authors:** Jian Yu, Dahang Zhao, Shuo Wang, Chao Zhang, Jiazhang Huang, Xu Wang, Xin Ma

**Affiliations:** ^1^Department of Orthopedics, Huashan Hospital, Fudan University, Shanghai, China; ^2^Department of Orthopedics, Ruijin Hospital, Shanghai Jiaotong University, Shanghai, China

## Abstract

The implant design of the talar component for total ankle replacement (TAR) should match the surface morphology of the talus so that the replaced ankle can restore the natural motion of the tibiotalar joint and may reduce postoperative complications. The purpose of this study was to introduce a new 3D fitting method (the two-sphere fitting method of the talar trochlea with three fitting resection planes) to approximate the shape of the upper part of the talus for the Chinese population. 90 models of the tali from CT images of healthy volunteers were used in this study. Geometrical fitting and morphological measurements were performed for the surface morphology of the upper part of the talus. The accuracy of the two-sphere fitting method of the talar trochlea was assessed by a comparison of previously reported data. Parameters of the fitting geometries with different sizes were recorded and compared. Results showed that compared with previously reported one-sphere, cylinder, and bitruncated cone fitting methods, the two-sphere fitting method presented the smallest maximum distance difference, indicating that talar trochlea can be approximated well as two spheres. The radius of the medial fitting sphere *R*_*M*_ was 20.69 ± 2.19 mm which was significantly smaller than the radius of the lateral fitting sphere *R*_*L*_ of 21.32 ± 1.88 mm. After grouping all data by the average radius of fitting spheres, the result showed that different sizes of the upper part of the talus presented significantly different parameters except the orientation of the lateral cutting plane, indicating that the orientation of the lateral cutting plane may keep consistent for all upper part of the talus and have no relationship with the size. The linear regression analyses demonstrated a weak correlation (*R*^2^ < 0.5) between the majority of parameters and the average radius of the fitting spheres. Therefore, different sizes of the upper part of the talus presented unique morphological features, and the design of different sizes of talar components for TAR should consider the size-specific characteristics of the talus. The parameters measured in this study provided a further understanding of the talus and can guide the design of different sizes of the talar components of the TAR implant.

## 1. Background

The kinematics of the tibiotalar joint during the gait is highly influenced by the surface morphology of ankle bones [1]. Thus, the implant design for total ankle replacement (TAR) should be based on the surface morphology of the tibia and talus, so that the replaced ankle can restore the natural motion of the tibiotalar joint and may reduce postoperative complications [2–5]. The implantation of the talar component of the TAR implant requires the resection of the proximal part of the talus. Therefore, investigation of the surface morphology of the upper part of the talus, especially the talar trochlea, is essential to the design of the talar component of the TAR implant. However, the design of the talar component varies among existing implant systems, and no consensus has been established [2, 6].

Shape approximation is one of the useful methods to study bone morphology and design ankle implants. Although several studies have measured the morphology of the talar trochlea [7–12], only a few studies [13–16] approximated the shape of the talar trochlea from the perspective of TAR implant design. Note that the majority of studies analyzed the shape of the talus in CT images considering that the thickness of the articular cartilage layer of the talar trochlea was shown in the previous literature [17] to be small and vary slightly, averaging 1.35 ± 0.22 mm in males and 1.11 ± 0.28 in females. Therefore, not including the articular cartilage layer in measuring models derived from CT images would produce limited errors.

**Figure 1:**
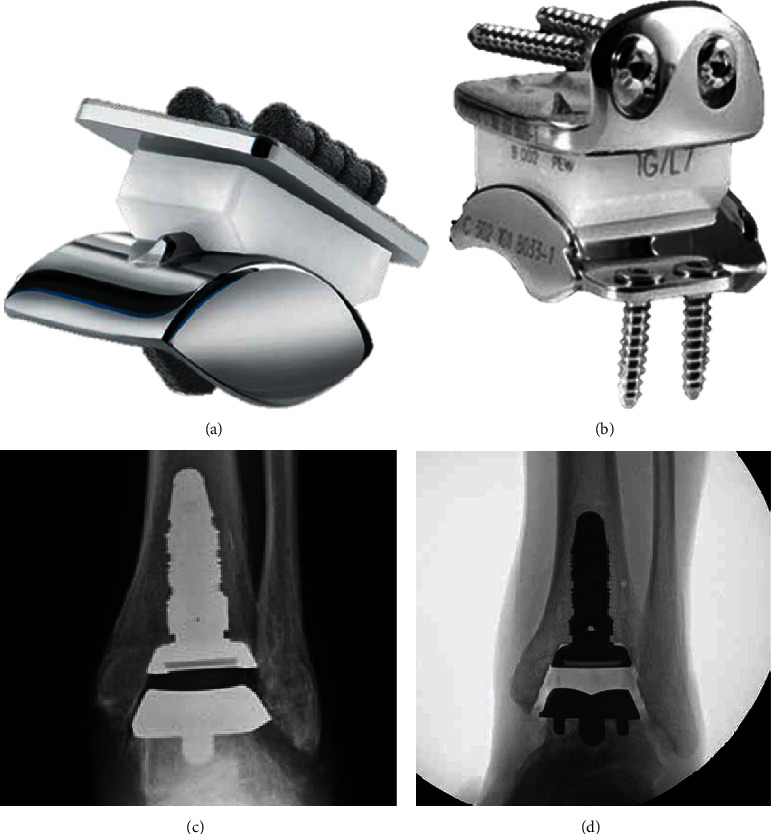
(a) Photograph of the Scandinavian Total Ankle Replacement (image courtesy of Stryker, Kalamazoo, MI, US). (b) Photograph of Hintegra total ankle prosthesis (image courtesy of Integra LifeSciences, Plainsboro, NJ, US). (c) Postoperative anterior-posterior radiograph of the INBONE ankle replacement (image courtesy of INBONE Technologies, Boulder, CO, US). (d) Postoperative anterior-posterior radiograph of the INBONE II ankle replacement (image courtesy of Wright Medical Group, Memphis, TN, US.).

Pioneered work by Inman [18] measured the shape of the talar trochlea of cadaver specimens and found out that the talar trochlea can be approximated as a truncated cone with its axis oriented from medial-distal end to lateral-proximal end. Although concerns have been raised, the concept of a truncated cone with one fixed axis has been used in the design of several early implant systems, such as the STAR implant system (Figure 1(a)) and the Hintegra implant system (Figure 1(b)). Implant systems with such designs have a natural instability along the medial/lateral direction, which requires additional central ridge or edge rims to constrain the medial/lateral motion of the bearing. However, unknown biomechanical risks might be brought by these structures for stabilization. To further approximate the shape of the talar trochlea and avoid additional structures for stabilization, some researchers considered the talar trochlea as a saddle-shaped truncated cone [8, 19]. Few implant systems, such as INBONE Total Ankle System (INBONE Technologies, U.S.) (Figure 1(c)), adopted this design. But the saddle-shaped talar component of INBONE Total Ankle System was later revised to INBONE II Total Ankle System (INBONE Technologies, U.S.) (Figure 1(d)) due to the high implant failure rate [2]. It may be explained by the fact that the articular surface of the latter implant system with the shape of two intersected spheres has a deeper sulcus in the center of the articular surface, thus providing higher stability of the implant system.

Fitting the medial and lateral surfaces of the talar trochlea with two spheres seems to be a good way to approximate the surface of the talar trochlea. However, in previous literature, this two-sphere fitting method has only been used to define the rotational axis of the tibiotalar joint [20, 21]. To define a reliable 3D modeling method for quantifying the shape of the upper part of the talus of the Chinese population, the present study approximated the shape of the talar trochlea using the two-sphere fitting method and the medial and lateral surfaces using the plane fitting method. The key parameters of these fitting geometries, including the radius of the spheres, the position of sphere origins, the inclination angle, and the position of the medial and lateral surfaces, were recorded. The accuracy of the two-sphere fitting method was assessed by a comparison of previously reported data. Also, the size difference of fitting geometries was further investigated to guide the design of the implant components with different sizes. We hypothesized that the parameters of the fitting geometries have a strong relationship with the size of the upper part of the talus.

## 2. Methods

### 2.1. Subjects

The protocol of this study was approved by the Ethics Committee of Huashan Hospital, Fudan University. The CT scan data of 90 intact ankles from previous studies were used to approximate the shape of the talar trochlea (46 males, 44 females, 23.47 ± 2.63 years, 19-37 years of age range, 171.49 ± 7.74 cm of height, and 66.00 ± 12.78 kg of body weight, 48 left ankles and 42 right ankles) [22, 23]. No a priori power analysis was performed since there were no data available to perform such analysis. CT examination was performed on a CT scanner (Brilliance iCT, Philips, Cleveland, U.S.) with 120 kV of voltage, 250 mA of current, 0.625 mm of slice thickness, and 512 × 512 pixels of matrix. The assessments of all CT images were performed by senior foot and ankle surgeons (X.W. and X.M.), and no signs of previous trauma, severe deformity, or degenerative changes in the ankle, such as ankle arthritis, osteochondral lesions of the talus, and talar cyst, were observed in the CT images of all ankles.

### 2.2. 3D Model Reconstruction

The Digital Imaging and Communications in Medicine (DICOM) file of each ankle was imported into Mimics (Materialise NV, Belgium) for the segmentation of the talus with a threshold of more than 203 Hounsfield units (HU) [24]. The 3D model of each talus was imported into 3-Matic Medical (Materialise NV, Belgium) for shape approximation and surface morphology measurement of the talar trochlea.

### 2.3. Shape Approximation of the Talar Trochlea

**Figure 2:**
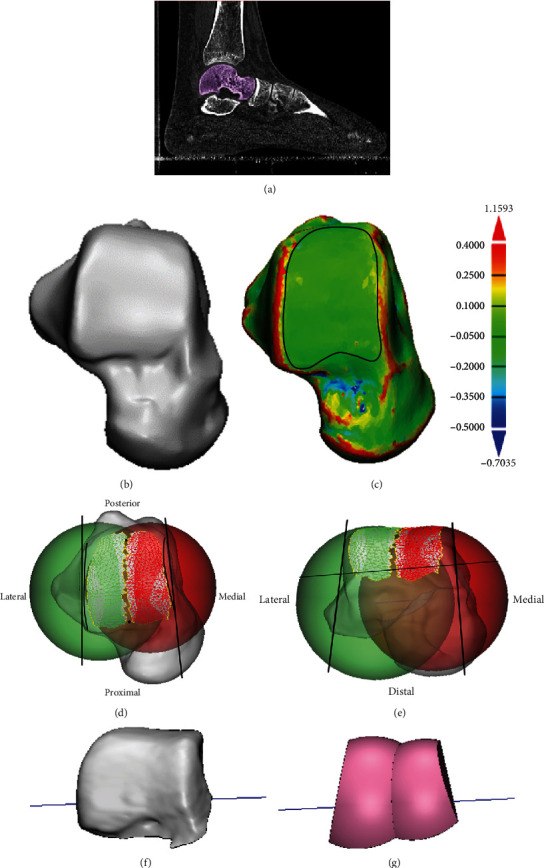
An illustration of the model reconstruction process and 3D fitting method. (a) Segmentation of the talus from CT images. (b) Acquiring the 3D model of the talus. (c) Curvature analysis and mesh selection. (d, e) The top (d) and front (e) views of the talus with the fitting two spheres, medial and lateral fitting plane, and horizontal plane. (f, g) Remaining bone fragment (f) and 3D fitting model (g) after the resection of the medial, lateral, and horizontal planes.

The two-sphere fitting method was described in previous studies [20, 21] to determine the rotation axis of the talus. We tried to apply this method to approximate the surface shape of the talar trochlea. This method used spheres to least-squares fit the medial or lateral part of the trochlear surface of the talus. The medial or lateral part of the trochlear surface of the talus was defined and manually selected as the facet surface between the central trochlea groove and the medial or lateral rim based on a curvature analysis of each talus. The curvature analysis built in the 3-Matic Medical (Materialise NV, Belgium) projected a color map of the surface curvature on the talar model which helped us identify the edge of each selection. The radius of the lateral sphere *R*_*L*_, the radius of the medial sphere *R*_*M*_, and the distance between sphere origins *L* were recorded. To define the size of each talar trochlea, we used the average radius which was the mean of *R*_*L*_ and *R*_*M*_ of each talus. The model reconstruction process and sphere fitting method are illustrated in Figure 2.

### 2.4. Coordinate System Definition

The method of a coordinate system definition was based on the subject-specific bone geometry of the talus and similar to that previously described by Yamaguchi et al. [25]. For each talus, the medial-lateral axis (*y*-axis) was defined as the line connecting the sphere origins (Point *O*_*L*_ and *O*_*M*_). The intersection of two spheres created an intersection circle. The origin of the coordinate system was defined as the center of the intersection circle, and the sagittal plane was defined as the intersection plane of the two spheres. The most anterior and posterior points of the medial and lateral edges of the talar trochlea were defined, and the midpoints of the most anterior or posterior points of both edges of the talar trochlea were calculated (Points *P*_*A*_ and *P*_*P*_). Then, a horizontal plane (plane *S*_*H*_) perpendicular to the sagittal plane which contained these two midpoints was created, and the superior-inferior axis (*z*-axis) was defined as the line perpendicular to the horizontal plane passing through the origin. The height (*H*) of the plane *S*_*H*_ was recorded. The anterior-posterior axis (*x*-axis) was a cross product of the superior-inferior and medial-lateral axes. Therefore, the coronal plane was formed by the intersection of the *x*-axis and *z*-axis, while the sagittal plane was formed by the intersection of the *y*-axis and *z*-axis. The Cartesian coordinate system was established in 3-Matics Medical (Materialise NV, Leuven, Belgium).

### 2.5. Shape Approximation of the Medial and Lateral Articular Surfaces

**Figure 3:**
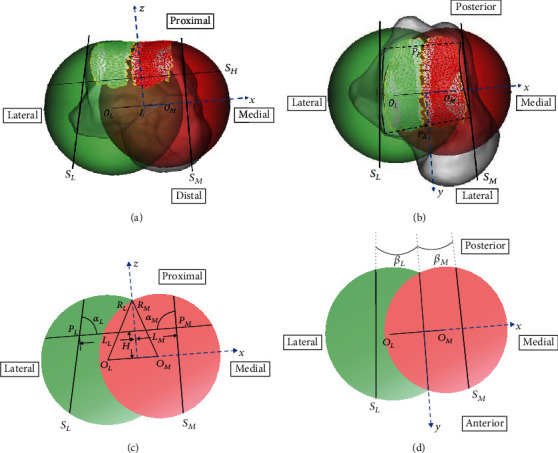
Establishment of the coordinate system ((a) front view and (b) top view of three-dimensional (3D) illustration) and geometry parameters of the talar trochlea ((c) front view and (c) top view of two-dimensional (2D) illustration. Parameters included the radius of the lateral sphere (*R*_*L*_), the radius of the medial sphere (*R*_*M*_), the distance between sphere origins (*L*), the height (*H*) of the plane *S*_*H*_, the position of planes *S*_*M*_ and *S*_*L*_ (*L*_*M*_ and *L*_*L*_), and the angles between the fitting planes to the horizontal or sagittal plane (*α*_*M*_, *α*_*L*_, *β*_*M*_, and *β*_*L*_)).

The medial or lateral articular surfaces of the upper part of the talus were defined and manually selected as the facet surface between the medial or lateral rim of the talus and the horizontal plane. The implantation of the talar component for total ankle replacement only requires the resection of the upper part of the talus. So, each talus was resected by the previously defined horizontal plane, and the medial and lateral articular surfaces can be fitted by two fitting planes (planes *S*_*M*_ and *S*_*L*_). To define these two fitting planes, the position of intersection points (Point *P*_*M*_) among the coronal plane, horizontal plane, and *S*_*M*_ was calculated (*L*_*M*_), while the intersection point (Point *P*_*L*_) among the coronal plane, horizontal plane, and *S*_*L*_ was also calculated (*L*_*L*_). The angles between the fitting plane to the horizontal or sagittal plane were also recorded (*α*_*M*_, *α*_*L*_, *β*_*M*_, and *β*_*L*_). The establishment of the coordinate system and geometry parameters of the talar trochlea were measured in 3-Matics (Materialise NV, Leuven, Belgium) and presented in Figure 3.

### 2.6. Evaluating the Accuracy of the 3D Fitting Method and Statistical Analysis

To evaluate the accuracy of the current 3D fitting method, sensitivity tests were performed in Supplementary File 1 to assess the stability of both the two-sphere fitting method to the talar trochlea and the plane fitting method to the medial and lateral articular surfaces. Also, the Euclidean distances between vertices on the talar trochlear surface and the geometries of two spheres were calculated and compared with the reported value of the single sphere, cylinder, or bitruncated cone fitting method by Huang et al. [16].

All data were presented as mean values with standard deviation (SD). To investigate the size difference of each parameter, we chose to divide all data into 4 groups based on the mean and standard deviation of the average radius (*R*_*L*_ + *R*_*M*_/2). All key parameters (*R*_*M*_: radius of the lateral sphere; *R*_*L*_: radius of the lateral sphere; *L*: the distance between sphere origins; *H*: the height of the plane *S*_*H*_; *L*_*M*_: the position of the medial fitting plane; *L*_*L*_: the position of the lateral fitting plane; *α*_*M*_: the angle between the horizontal plane and the medial fitting plane; *α*_*L*_: the angle between the horizontal plane and the lateral fitting plane; *β*_*M*_: the angle between the sagittal plane and the medial fitting plane; *β*_*L*_: the angle between the sagittal plane and the lateral fitting plane) both overall or within each size group were checked for normal distribution and homogeneity of variance. A paired *t*-test was used to determine the significance of the difference between the radius of the medial and lateral spheres or between two poses. One-way repeated measures Analysis of Variance (ANOVA) was conducted to compare the distances or angles among each group. Statistical significance level *p* was set at 0.05. The linear regressions between the average radii and other parameters of the fitting models (*L*, *H*, *L*_*M*_, *L*_*L*_, *α*_*M*_, *α*_*L*_, *β*_*M*_, and *β*_*L*_) were performed, and the correlation coefficients (*R*^2^) were computed. Data were processed in MATLAB (2018b, MathWorks Inc., Natick, MA).

## 3. Results

The sensitivity analyses in Supplementary File 1 showed that changing the area of the selected trochlear surface of the talus (see Figure S1) made a small impact on the radius of the spheres and the position of the sphere origin (maximum difference of the radii of the medial and lateral fitting spheres was both less than 5%). Also, changing the area of the selected medial articular surfaces of the talus (Figure S2) made a small impact on the orientation of the medial fitting plane (the maximum difference of the inclination angle was less than 5%). However, due to the curved shape of the lateral articular surface, the inclination angle of the lateral fitting plane varied as the selection area changed. Therefore, the selection of the lateral articular surface should be carefully selected above the horizontal plane.

Compared with fitting methods of one sphere, cylinder, and bitruncated cone, the mean, standard deviation, and maximum of the distances between the talar trochlea and two-sphere model are given in Table 1 [16]. The two-sphere fitting method presented a relatively small mean and the smallest standard deviation and maximum (0.43 ± 0.06 mm, 0.23 ± 0.08 mm, and 1.41 ± 0.61 mm, respectively).

The mean and SD of all parameters grouped by the mean and SD of the average radius are listed in Table 2. The overall average radius was 21.00 ± 2.09 mm. Thus, the four groups were average radius < 18.91 mm, average radius > 18.91 and <21.00 mm, average radius > 21.00 and <23.09 mm, and average radius > 23.09 mm. All values of the geometry parameters of the talar trochlea (*R*_*M*_, *R*_*L*_, *L*, *H*, *L*_*M*_, *L*_*L*_, *α*_*M*_, *α*_*L*_, *β*_*M*_, *β*_*L*_) are presented in Supplementary File 2, and they were normally distributed in both overall and within each size group category. Also, the variance of each parameter was not significantly different. The radius of the medial fitting sphere *R*_*M*_ was 20.69 ± 2.19 mm which was significantly smaller than the radius of the lateral fitting sphere *R*_*L*_ of 21.32 ± 1.88 mm (*p* < 0.05). Statistically significant differences among four groups were found in *R*_*M*_, *R*_*L*_, *L*, *H*, *L*_*M*_, *L*_*L*_, *α*_*M*_, *α*_*L*_, *β*_*M*_, and *β*_*L*_. The mean of *R*_*M*_, *R*_*L*_, *L*, *H*, *L*_*M*_, *L*_*L*_, and *β*_*M*_ increased with the average radius, while the mean of *α*_*M*_ decreased with the average radius.

**Figure 4:**
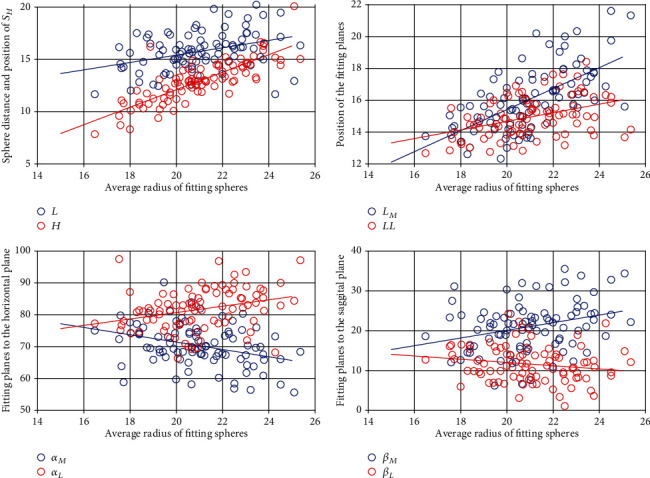
Plots of the results of linear regressions between the average radii and other parameters of the fitting models (*L*, *H*, *L*_*M*_, *L*_*L*_, *α*_*M*_, *α*_*L*_, *β*_*M*_, and *β*_*L*_).

The result of linear regressions between the average radii and other parameters of the fitting models is illustrated in Figure 4, and the correlation coefficients for *L*, *H*, *L*_*M*_, *L*_*L*_, *α*_*M*_, *α*_*L*_, *β*_*M*_, and *β*_*L*_ were 0.1376, 0.6534, 0.4074, 0.1706, 0.1116, 0.0871, 0.0864, and 0.0294, respectively.

## 4. Discussion

In this study, we proposed a method for shape approximation of talar trochlea with two spheres resected by three fitting planes and investigated the size difference of the parameters for this fitting method (Figure 3). Calculation of the distances between the surface of the talar trochlea and the two-sphere model showed that the current fitting method was an accurate shape approximate method to the talar trochlea. The sensitivity test also showed that fitting the talar trochlea with two spheres was a stable method with a small selection bias.

Since the mean and standard deviation of the distance difference for all fitting methods were below the slicing distance of the CT scan, the maximums of the distances between vertices on the talar trochlea and geometrical models were primarily used to assess the accuracy of the fitting method. Compared with other fitting methods, the two-sphere fitting method presented the smallest maximum (1.41 ± 0.61 mm), suggesting that the similarity between the talar trochlea and the two-sphere fitting method was more prominent and this method seems to be a reasonable choice for shape approximation of the talar trochlea.

Several studies have previously reported the radii of the talar trochlea, and their results are listed in Table 3 [7–9, 15, 22, 26, 27]. The disagreements on the measurement of the medial and lateral curvature radii of the talar trochlea seem to be well explained by the truncated cone model presented by Inman [18]. The cross-sections of a cone intersected by two planes can be two ellipses. Therefore, the medial and lateral radii of curvature of the talar trochlea may not be equal. Similarly, the anterior and posterior radii of curvature of one side of the talar trochlea may not be the same. Modified from the cone fitting method, the two-sphere fitting method shared the rotational axis of the cone but provided a closer shape approximation at the central groove and medial and lateral rims. The average and standard deviation of the radii of the talar trochlea measured in this study were in the range of previously reported values [7–9, 15, 22, 26, 27].

However, the radii measured in this study showed a high variety, indicating high anatomical variability of the size of talar trochlea in the Chinese population. The ranges of the medial and lateral radii were 16.39 to 26.27 degrees and 16.46 to 25.18 degrees, respectively. Although statistical analysis showed that the radius of the lateral fitting sphere was found to be significantly larger than that of the medial fitting sphere, 27 out of 90 tali (30%) had a larger medial sphere radius *R*_*M*_. Therefore, the design of the talar component for total ankle replacement should prepare extra configuration for the need of special cases.

Only one of the existing total ankle implant systems (Cadence implant system, Integra Lifesciences, Cordova, TN, U.S.) had different medial and lateral sphere radii in the design of talar component for TAR with 8 degrees of conical angle, and the lateral sphere is larger than the medial side. This value was derived from the mean angle between the axis and the horizontal plane of the ankle measured by Inman [18]. The current study also found that the lateral sphere presented a statistically significantly larger mean radius than the medial sphere. The mean conical angle of the current study can be calculated from the value of mean lateral radius ($\bar{R_{L}}$), mean medial radius ($\bar{R_{M}}$), and the mean distance between sphere origins ($\bar{L}$) as $\arcsin\left(\;\left|\bar{R_{M}}-\bar{R_{L}}\right|/\bar{L}\right)$, which was 2.29 degrees. The difference between these may be due to the population difference.

After grouping all data by the average radius of fitting spheres, the result showed that different sizes of the upper part of the talus presented significantly different parameters except the orientation of the lateral cutting plane, indicating that the orientation of the lateral cutting plane may keep consistent for all upper part of the talus and have no relationship with the size. The result of the linear regression showed that the majority of parameters showed a weak relationship (less than 0.5) to the average radius except for the height of *S*_*H*_ with a value of 0.6534. *S*_*H*_ was determined by the medial and lateral edges of the talar trochlea which were strongly related to the medial and lateral radii. Small *R*^2^ also rejected our conjecture that parameters of the fitting geometries have a strong relationship with the size of the upper part of the talus. Therefore, the design of the different sizes of talar components for TAR should consider the size-specific characteristics of the talus instead of simply scaling the shape of one general model.

There were some limitations of the current study. First, the sample size of this study is limited. Although our sample size was larger than the majority of previous studies [7–9, 15, 22, 26, 27], future studies should recruit a large number of subjects to increase the power of the study and expand the participants to older population or patients with damaged talar trochlea to improve the clinical relevance. Second, the two-sphere fitting method presented here for shape approximation only studied the surface of the talus. The mated surfaces of the tibia and fibula within the tibiotalar joint and the anatomy of the tibiofibular syndesmosis should also be considered in future studies. The kinematics of the tibiotalar should also be considered. Future studies should evaluate whether the axis by connecting the fitting sphere origins was the rotational axis of the tibiotalar joint during the gait. Third, the two-sphere fitting method proposed in the current study has not been proved as the best method for talar shape approximation with the most accuracy. Future studies should compare the two-sphere fitting method with more new geometric fitting methods to better approximate the morphology of the talus. Last, the current fitting geometry of two spheres with three resection planes is only the first step of implant design for TAR. Before a finished product, additional manufacturing processes will be required, such as fillets will be added to the edges to obtain smooth transitions from surfaces to surfaces to avoid shape edges of the fitting geometry. Still, the results of this study could be a useful reference for implant component design for TAR.

## 5. Conclusion

The two-sphere fitting method of the talar trochlear was a stable and accurate shape approximation method with the smallest difference of maximum distance. The medial fitting sphere has a significantly smaller radius than the lateral fitting sphere. The presented fitting method with two spheres and three resection planes can be used as a standardized definition for shape approximation of the upper part of the talus. Also, different sizes of the upper part of the talus presented unique morphological features, and the design of different sizes of talar components for TAR should consider the size-specific characteristics of the talus. The parameters measured in this study provided a further understanding of the talus and can guide the design of different sizes of the talar components of the TAR implant.

## Figures and Tables

**Table 1:** The mean, standard deviation, and maximum of the distances between vertices on the talar trochlea and geometrical models of one sphere, cylinder, bitruncated cone, and two spheres.

Fitting method	Sample size	Distance (mm)		
		Mean	Standard deviation (SD)	Maximum
Single sphere (Huang et al.)	50	1.16 ± 0.21	0.73 ± 0.13	3.55 ± 0.77
Single cylinder (Huang et al.)	50	0.44 ± 0.08	0.40 ± 0.08	2.81 ± 0.72
Bitruncated cone (Huang et al.)	50	0.36 ± 0.07	0.32 ± 0.06	2.24 ± 0.47
Bitruncated cone (Kleipool et al.)-RMSE	40	0.41 ± 0.09	N.R.	N.R.
Two spheres (current study)	90	0.43 ± 0.06	0.23 ± 0.08	1.41 ± 0.61

^∗^N.R.: not reported.

**Table 2:** Key parameters of fitting geometries in different size groups.

	Mean	Range	Group by the average radius ± one SD				
			<18.91 mm	>18.91 and <21.00 mm	>21.00 and <23.09 mm	>23.09 mm	Significance of difference among groups
*N* ^∗^	—	—	14	34	29	13	—
*R*_*M*_ (mm)	20.69 ± 2.19	16.39-26.27	17.71 ± 0.38	20.30 ± 0.45	22.75 ± 0.71	23.93 ± 1.00	*p* < 0.05
*R*_*L*_ *(mm)*	21.32 ± 1.88	16.46-25.18	18.68 ± 0.55	21.14 ± 0.53	22.65 ± 0.84	24.09 ± 0.62	*p* < 0.05
*L* (mm)	15.76 ± 1.83	11.67-20.23	15.12 ± 1.47	15.36 ± 1.17	16.89 ± 1.10	16.28 ± 1.90	*p* < 0.05
*H* (mm)	12.94 ± 1.99	7.85-20.80	10.86 ± 1.17	12.95 ± 0.46	13.99 ± 0.74	15.48 ± 1.24	*p* < 0.05
*L*_*M*_ (mm)	16.07 ± 1.99	12.33-21.61	14.45 ± 0.86	15.46 ± 1.09	17.71 ± 1.39	18.02 ± 1.42	*p* < 0.05
*L*_*L*_ (mm)	14.95 ± 1.26	12.68-18.42	14.09 ± 0.69	14.80 ± 0.86	15.58 ± 1.09	15.54 ± 1.16	*p* < 0.05
*α*_*M*_ (degrees)	70.26 ± 6.65	55.61-90.18	72.88 ± 4.65	71.39 ± 4.32	69.21 ± 6.26	65.49 ± 5.03	*p* < 0.05
*α*_*L*_ (degrees)	81.69 ± 6.55	66.35-97.50	80.64 ± 4.12	80.16 ± 4.80	85.27 ± 4.29	83.66 ± 5.13	*p* < 0.05
*β*_*M*_ (degrees)	21.05 ± 6.30	6.21-35.52	19.05 ± 5.33	19.46 ± 4.85	22.32 ± 5.56	25.28 ± 4.71	*p* < 0.05
*β*_*L*_ (degrees)	11.61 ± 4.57	1.07-24.27	12.65 ± 2.94	11.82 ± 3.93	8.87 ± 2.99	11.33 ± 3.44	*p* > 0.05

^∗^ *N*: number of subject in each group.

**Table 3:** Summary of the radii of the talar trochlea measured from previously published data.

Authors+published year	Sample size	Image source	Radii of the medial curvature of the talar trochlea (mm)	Radii of the medial curvature of the talar trochlea (mm)	Overall radii of the talar trochlea (mm)
Stagni et al. 2005	21	X-ray	—	—	23.4 ± 3.1
Hayes et al. 2006	36	CT	—	—	20.7 ± 2.6
Wiewiorski et al. 2012	83	CT	20.4 ± 2.5	20.3 ± 2.0	—
Siegler et al. 2014	26	CT	25.7 ± 4.8	21.7 ± 2.9	—
Kuo et al. 2014	50	CT	21.8 ± 2	20.8 ± 3	—
Nozaki et al. 2016	58	CT	14.7 ± 1.8 (anterior)	22.5 ± 2.4 (anterior)	—
			24.0 + 4.1 (posterior)	23.3 + 3.4 (posterior)	
Zhao et al. 2019	71	CT	17.02 ± 3.49 (anterior)	19.23 ± 2.47 (anterior)	—
			22.75 ± 3.04 (posterior)	18.76 ± 2.90 (posterior)	
Current study	90	CT	20.69 ± 2.19	21.32 ± 1.88	—

## Data Availability

The datasets used and analyzed during the current study are available from the corresponding author on reasonable request.

## Supplementary Materials

Supplementary Materials The present study included two supplementary files, which were all referred to in the manuscript.
