# Supplementary material for: Shape Approximation and Size Difference of the Upper Part of the Talus: Implication for Implant Design of the Talar Component for Total Ankle Replacement
Source: Biomed Res Int. 2022 Jan 12;2022:1248990. doi: 10.1155/2022/1248990 (PMC8769841; doi:10.1155/2022/1248990)
Supplement: Supplementary Materials — The present study included two supplementary files, which were all referred to in the manuscript. [file 1248990.f1.zip › Supplementary File 1.docx]

Supplementary File 1

1. Sensitivity analysis of the selection on the surface of talar trochlea for sphere fitting

A sensitivity analysis of the selection on the surface of talar trochlea for sphere fitting on one of the subjects was performed to assess the stability of the two-sphere fitting method. First, manually selected the facet surface between the central trochlea groove and the medial or lateral rim. Then, shrink the selection by one mesh from the edge to the center. Last, sphere fitting each selection. (The sensitivity analysis was illustrated in **Fig. S1）**


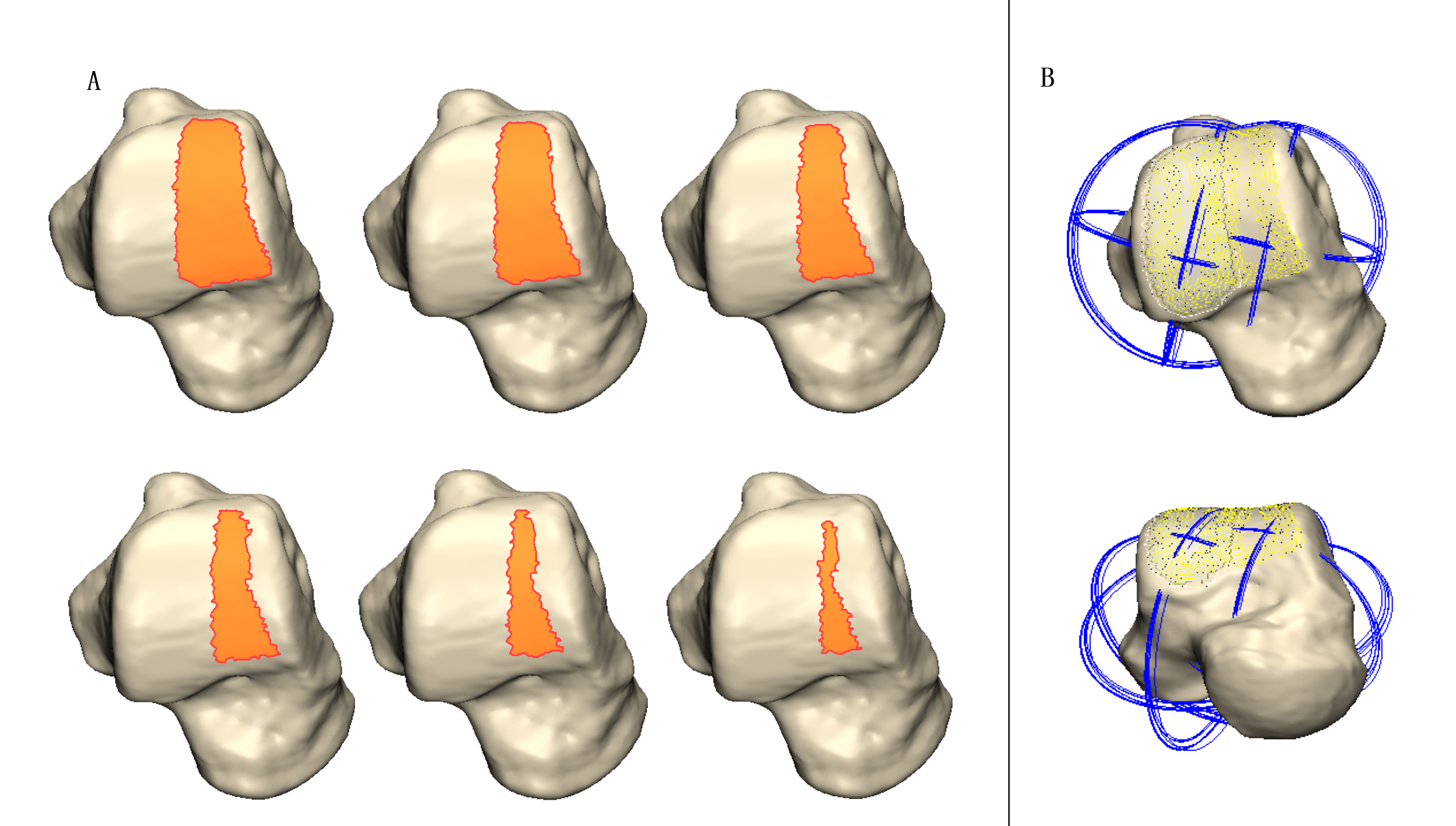


**Figure S1** an illustration of the sensitivity analysis of the area selection on the surface of talar trochlea for sphere fitting. (A) Shrinking the area of selection (Take medial facet of the talar trochlea as an example); (B) Sphere fitting each area selected.

The result for the sensitivity analysis was listed in **Table S1**. The positions of the sphere origins and the radii of the medial or lateral fitting sphere were not sensitive to the selection area. The maximum difference among different areas selected for medial and lateral spheres (from 87.64$\mathrm{mm}^{2}$ to 441$\mathrm{mm}^{2}$ and from 59.68$\mathrm{mm}^{2}$ to 553.00$\mathrm{mm}^{2}$, respectively) were 4.04% and 4.39%, respectively, which were both less than 5%.

**Table S1** Sensitivity analysis of the selection on the surface of talar trochlea for sphere fitting.

|  | Area ($\mathrm{mm}^{2})$ | Number of meshes | Number of nodes | Radius (mm) | Coordinates of sphere origins |
| --- | --- | --- | --- | --- | --- |
| Medial Surface 1 | 441.00 | 1594 | 869 | 20.59 | 111.65，151.10，267.24 |
| Medial Surface 2 | 363.75 | 1327 | 728 | 20.03 | 111.36，151.54，267.26 |
| Medial Surface 3 | 289.20 | 1076 | 600 | 19.84 | 111.22，151.62，267.14 |
| Medial Surface 4 | 218.01 | 822 | 475 | 20.73 | 110.53，150.65，266.71 |
| Medial Surface 5 | 149.03 | 567 | 340 | 19.98 | 110.97，151.92，267.33 |
| Medial Surface 6 | 87.64 | 344 | 222 | 19.82 | 110.86，151.36，266.58 |
| The maximum difference of the radii of the medial fitting spheres： | | | | 4.39% | |
| Lateral Surface 1 | 553.00 | 1877 | 1011 | 22.10 | 117.24，149.58，280.89 |
| Lateral Surface 2 | 472.07 | 1599 | 868 | 22.90 | 117.64，148.68，281.14 |
| Lateral Surface 3 | 390.27 | 1311 | 729 | 22.99 | 117.71，148.52，281.28 |
| Lateral Surface 4 | 310.23 | 1037 | 583 | 22.11 | 117.40，149.34，281.40 |
| Lateral Surface 5 | 236.61 | 794 | 455 | 22.86 | 117.60，148.49，281.33 |
| Lateral Surface 6 | 170.62 | 579 | 340 | 22.16 | 117.28，149.12，281.20 |
| Lateral Surface 7 | 112.47 | 382 | 240 | 22.66 | 117.25，148.54，280.81 |
| Lateral Surface 8 | 59.68 | 202 | 143 | 23.03 | 116.89，148.12，279.56 |
| The maximum difference of the radii of the lateral fitting spheres： | | | | 4.04% | |

2. Sensitivity analysis of the selection on the medial and lateral articular surface of the talus for plane fitting

To define the medial and lateral resection plane, the medial and lateral articular surfaces of the talus were fitting by planes. A sensitivity analysis of the selection on the medial and lateral articular surface of the talus for plane fitting was performed. First, we manually selected the medial or lateral surface between the edge of the articular surface and the medial or lateral rim. Then, gradually adjust the selection until all meshs selected were above the horizontal resection plane. Last, use plane to fit each selection. (The sensitivity analysis was illustrated in **Figure S2 and S3）**


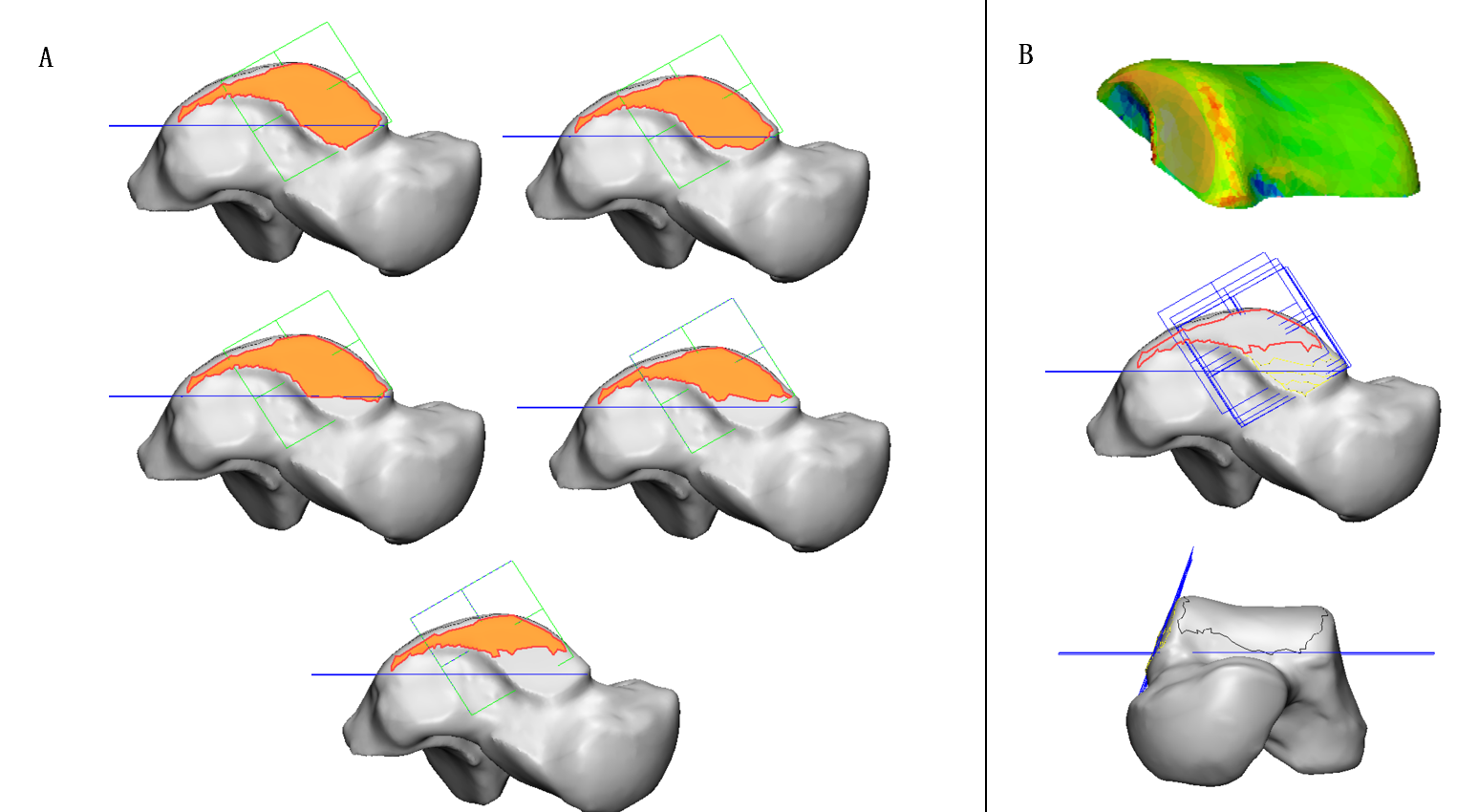


**Figure S2** an illustration of the sensitivity analysis of the area selection on the medial articular surface of the talus for plane fitting. (A) Adjusting the area of selection; (B) Plane fitting each area selected.


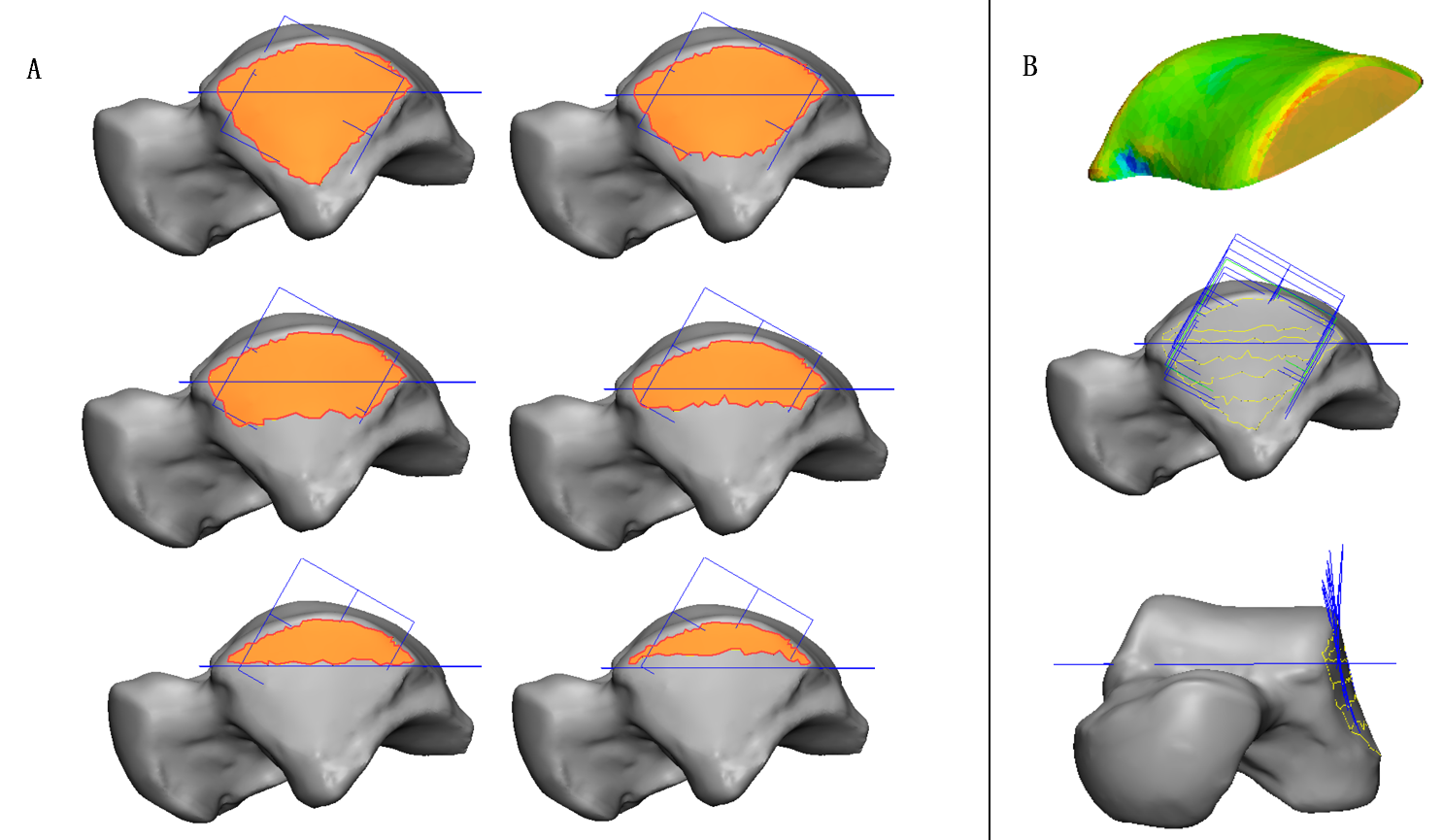


**Figure S3** an illustration of the sensitivity analysis of the area selection on the lateral articular surface of the talus for plane fitting. (A) Adjusting the area of selection; (B) Plane fitting each area selected.

The result for the sensitivity analysis was listed in **Table S2**. The angle of the medial fitting plane to the horizontal plane were not sensitive to the selection area. The maximum difference among different areas selected for medial articular surface (68.31 to 71.02 degrees from the area of 111.2 $\mathrm{mm}^{2}$ to 200.25 $\mathrm{mm}^{2}$) were 3.82%, which were less than 5%. However, the angle of the lateral fitting plane to the horizontal plane increased with the area of the the selection. The maximum difference among different areas selected for lateral articular surface (75.34 to 93.38 degrees from the area of from 98.98 $\mathrm{mm}^{2}$ to 386.85$\mathrm{mm}^{2}$) were 19.25%, which was quite large. Therefore, when selecting the lateralarticular surface, be careful not to incorrectly select the region below the horizontal resection plane.

**Table S2** Sensitivity analysis of the selection on the medial and lateral articular surface of the talus for plane fitting.

|  | Area ($\mathrm{mm}^{2})$ | Number of meshes | Number of nodes | Angle to the horizontal plane (degrees) |
| --- | --- | --- | --- | --- |
| Medial articular Surface 1 | 200.25 | 312 | 202 | 68.93 |
| Medial articular Surface 2 | 192.68 | 287 | 186 | 68.31 |
| Medial articular Surface 3 | 177.96 | 270 | 176 | 69.58 |
| Medial articular Surface 4 | 149.75 | 234 | 156 | 70.36 |
| Medial articular Surface 5 | 111.20 | 193 | 132 | 71.02 |
| The maximum difference of the angle | | | | 3.82% |
| Lateral articular Surface 1 | 386.85 | 572 | 330 | 75.34 |
| Lateral articular Surface 2 | 349.85 | 494 | 289 | 76.99 |
| Lateral articular Surface 3 | 288.20 | 404 | 240 | 80.90 |
| Lateral articular Surface 4 | 223.22 | 309 | 191 | 82.78 |
| Lateral articular Surface 5 | 141.96 | 187 | 124 | 84.78 |
| Lateral articular Surface 6 | 98.98 | 140 | 101 | 93.38 |
| The maximum difference of the angle | | | | 19.25% |
