# Supplementary material for: Shape Approximation and Size Difference of the Upper Part of the Talus: Implication for Implant Design of the Talar Component for Total Ankle Replacement
Source: Biomed Res Int. 2022 Jan 12;2022:1248990. doi: 10.1155/2022/1248990 (PMC8769841; doi:10.1155/2022/1248990)
Supplement: Supplementary Materials — The present study included two supplementary files, which were all referred to in the manuscript. [file 1248990.f1.zip › Supplementary File 2.pdf]

Supplementary File 2

Group	Subject No	Age	Is Female	Is Left Side	R of the medial sphere	R of the lateral sphere	Average Radius	L	H	L_M	L_L	α_M	α_L	β_M	β_L
1	1	22	1	1	16.49	16.458	16.474	11.67	7.85	13.7	12.7	75	77.3	18.6	12.7
1	2	22	1	0	17.56	17.49	17.525	16.17	9.6	15.1	12.9	72.4	97.5	23.3	16.1
1	3	22	1	0	16.62	18.55	17.585	14.57	8.71	13.8	15.5	79.8	75.1	12	16.4
1	4	22	1	0	17.46	17.8	17.63	14.16	11.1	14.3	13.6	63.8	76.7	27.5	13.9
1	5	22	1	0	17.13	18.31	17.72	14.26	10.8	14.4	14	58.8	78	31.1	12.9
1	6	22	1	1	18.04	17.95	17.995	15.62	8.32	16	13	77.6	87.2	13	5.94
1	7	22	1	1	17.66	18.4	18.03	12.02	10.1	13.4	13.2	74.3	74.2	23.9	16.5
1	8	23	1	0	17.62	18.96	18.29	17.52	12	12.6	14.9	77.1	80.8	14.5	9.92
1	9	21	1	1	18.09	18.7	18.395	12.82	10.7	15.6	14.5	73.8	74.7	16.2	15.4
1	10	24	1	0	17.69	19.1	18.395	13.65	10.9	13	13	74.2	81.4	14	16.5
1	11	22	1	1	17.52	19.62	18.57	14.77	10.3	14	14	76	81.1	16	9.86
1	12	22	1	1	18.2	19.25	18.725	17.09	11.7	15	15	69.7	78.4	20	12.4
1	13	22	1	1	17.85	19.75	18.8	17.39	10.7	14.7	14.3	80.5	82.1	12.8	9.61
1	14	22	1	1	18.85	18.9	18.875	16.51	16.2	16.4	14.9	69.5	81.3	23	9.06
2	15	22	1	1	18.49	19.52	19.005	12.59	11.3	16	14	65.9	78.5	25	11.7
2	16	20	1	1	18.55	19.89	19.22	12.45	9.39	15	14	73	83.7	18	6.88
2	17	22	1	0	20.08	18.45	19.265	15.43	13.1	16.2	12.8	69.1	80.1	21	17.1
2	18	23	1	1	18.06	20.5	19.28	16.36	10.2	14.6	15.8	68.2	81.2	21.9	9.87
2	19	24	0	1	18.99	19.85	19.42	15.88	10.7	17.3	16	68.4	86.6	21.6	6.94
2	20	22	0	1	18.77	20.16	19.465	13.5	11.3	14	14	90.2	74.9	6.2	16
2	21	22	1	0	19.6	19.48	19.54	13.69	12.2	14.9	14.5	66.7	76.4	23.6	14.8
2	22	21	0	1	20.17	19.1	19.635	17.59	11	18	14	73.1	83.4	19	7.05
2	23	19	1	1	19.97	19.47	19.72	13.55	11.6	16	13	71.1	81.4	19	9.9
2	24	22	1	0	17.89	21.57	19.73	17.36	11.8	12.3	15.2	81.1	72.7	12.4	15.7
2	25	24	0	0	19.82	19.94	19.88	14.94	10.1	16.1	14.9	69.3	86.4	23.3	6.68
2	26	22	1	0	19.32	20.57	19.945	15.06	12.7	13.6	14.8	74.2	72.7	18.9	16.2
2	27	23	1	0	19.47	20.51	19.99	15.93	13.1	16.8	13.8	59.7	83	30.3	11.1
2	28	22	1	0	19.05	20.95	20	14.49	11.2	13	16.5	78	76.5	20	14.1
2	29	22	1	0	19.02	21.1	20.06	15.06	12.8	14.2	16.3	68.2	66.4	21.9	24.3

2	30	21	1	1	19.17	21.11	20.14	16.37	13.3	14	15	76.9	77.4	14	12.7
2	31	22	1	1	19.52	20.8	20.16	15.53	12.4	15	14	66	77.2	24	13.8
2	32	22	1	1	20.3	20.17	20.235	15.74	12	15	14	79	81	12	10.8
2	33	22	1	1	19.92	20.65	20.285	14.81	14	13.8	13.1	68.4	83	21.7	8.45
2	34	25	0	0	19.9292	20.8392	20.3842	14.53	10.7	15.4	16.9	78	70.9	12.1	19.2
2	35	23	1	0	18.48	22.56	20.52	17.29	14.1	13.5	16.4	62.1	70	28.4	22.3
2	36	23	0	1	21	20.05	20.525	15.98	12.9	17.2	14.5	65	88.2	25.9	3.08
2	37	23	1	0	19.85	21.28	20.565	15.56	13	13.9	13.9	74	83.6	16	8.07
2	38	24	1	1	20.1388	20.995	20.5669	15.58	12.5	15	15	74.5	77.9	15.4	13.6
2	39	22	1	1	21	20.31	20.655	12.59	12.9	19	14	58.2	78.4	32	11.7
2	40	22	0	1	19.43	21.88	20.655	15.96	13.1	15	17	83.9	69.3	6.6	20.8
2	41	25	1	0	20.656	20.684	20.67	14.31	13.8	14.5	13.8	69.9	83.2	20.2	9.34
2	42	22	0	1	20.96	20.4	20.68	14.06	12.3	16	13	69.8	82.3	22	14
2	43	31	0	0	20.266	21.12	20.693	15.71	14.9	16	15	68.2	85.7	24.9	6.45
2	44	24	0	0	20.6	20.99	20.795	17.99	13	16.1	16.1	69.2	81.3	22.3	10.9
2	45	24	1	0	19.5259	22.1919	20.8589	14.79	12.5	13.7	16	73.1	74.4	16.9	15.8
2	46	22	0	0	20.35	21.37	20.86	18.29	12.6	15.8	14.8	75.7	82.8	14.8	11.7
2	47	20	1	1	19.69	22.16	20.925	13.46	11.9	14	15.8	74.7	69.6	15	20.5
2	48	26	0	0	20.488	21.417	20.9525	15.41	12.9	15.3	14	71.8	85.2	20.8	7.75
3	49	23	1	0	20.07	21.99	21.03	16.07	13.4	13.6	14.2	81.9	84.1	7.57	8.37
3	50	23	1	1	20.03	22.07	21.05	16.82	14.1	14	15	67.7	71.7	24	18.7
3	51	25	1	1	21.64	20.55	21.095	15.61	11.9	17.7	15.5	67.7	81.3	22.4	9.9
3	52	33	0	1	20.8	21.41	21.105	17.76	12.6	17.3	15.4	69.9	90.1	21.4	6.51
3	53	23	0	0	20.6094	21.8181	21.21375	16.34	13	16.5	15.8	67.3	75.8	23.3	13.8
3	54	23	0	1	21.69	20.85	21.27	14.89	13	20.2	15.3	70	88	31.2	8.49
3	55	25	0	1	21.48	21.09	21.285	15.28	12.9	15.8	13.4	70.2	88.3	20	6.56
3	56	25	0	0	20.6309	22.6155	21.6232	19.83	13.2	17.5	17.2	65.7	78.2	24.4	14.1
3	57	26	0	1	21.31	21.99	21.65	15	12.4	15.7	14.5	71.3	89.1	19.6	7.43
3	58	22	1	0	20.94	22.53	21.735	16.5	14.5	14.6	15.3	67	86.8	23.2	11.2
3	59	24	1	0	21.48	22.15	21.815	15.53	14.5	15	15	68	77.3	23	15.5
3	60	24	0	1	22.23	21.44	21.835	16.16	14.4	19.5	15.2	60.2	96.9	31	12.4

3	61	23	0	0	21.88	22.1	21.99	17.25	13.9	16.6	17.6	79.7	89.4	10.9	5.26
3	62	29	0	1	21.1697	22.812	21.99085	13.61	13.1	16.6	14.6	67.7	82.6	22	10.4
3	63	27	0	0	21.74	22.45	22.095	18.11	13.4	16.6	15.5	73.4	77.6	16.7	13.4
3	64	26	0	1	22.6	21.67	22.135	16.49	15.2	16.7	13.7	67.4	88	23.2	14
3	65	25	0	1	22.4	22.11	22.255	19.34	14.8	16.2	15.1	74.7	79.5	17.6	13.2
3	66	25	0	1	22.32	22.24	22.28	14.82	11.7	19	16.9	74	86.7	18.1	3.36
3	67	22	0	0	21.56	23.31	22.435	16.37	12.9	17.9	17.6	78.1	81.8	14.7	9.54
3	68	24	0	0	23.04	21.85	22.445	16.23	14.4	18	15.2	72.5	87.9	20.7	9.12
3	69	22	1	0	23.46	21.54	22.5	15.29	13	19	13	56.8	90	36	1.07
3	70	21	1	1	23.84	21.18	22.51	16.02	13.9	20	14	58.4	92.6	32	7.38
3	71	37	0	0	22.0394	23.5302	22.7848	16.15	15.1	17	16.4	66.1	85.3	23.8	5.55
3	72	22	0	1	22.85	22.83	22.84	16.91	14.4	16.4	15.1	75.2	85.1	16.8	10.5
3	73	27	0	0	22.68	23.07	22.875	16.18	14.2	15.7	15.6	80.3	82.2	12.3	10.8
3	74	25	0	1	23.89	22.12	23.005	18.28	13.7	18.3	15.1	67.3	93.4	24.7	8.13
3	75	23	0	1	24.01	22.05	23.03	17.51	14.6	20.3	14.6	61.8	88.9	28	9.48
3	76	22	0	0	21.26	24.85	23.055	17.54	14.6	16	18	64.2	72	26	18.9
3	77	26	0	0	22.352	23.8237	23.08785	18.95	14.7	16.4	16.6	70.3	83.1	20	8.26
4	78	20	0	1	22.67	23.75	23.21	14.43	14.5	19	17	56.4	74.5	34	16.2
4	79	25	0	1	22.98	23.71	23.345	16.03	15.3	16.5	16.5	63.8	78.1	26.3	12
4	80	25	0	0	23	23.87	23.435	17.36	13.3	17.1	18.4	69.7	83.7	21	8
4	81	26	0	1	23.15	23.77	23.46	20.23	13.1	17.9	15	76	86.2	14.5	12.1
4	82	23	0	1	24.08	23.19	23.635	15.23	16.7	17.5	14.1	69.9	88.2	24.2	4.92
4	83	23	0	0	23.05	24.46	23.755	19.25	15.9	18	16	67.6	85.3	23	12.5
4	84	24	0	1	23.74	23.78	23.76	15	16.3	17.7	13.8	60.9	87.7	29.7	3.63
4	85	25	0	0	22.9969	24.5855	23.7912	16.5	16.6	16	14.9	65.8	81.3	24.4	10.8
4	86	26	0	0	23.367	25.18	24.2735	11.67	15.2	16.9	16.3	73.2	68.2	18.7	21.9
4	87	22	1	1	25.15	23.86	24.505	17.15	14.5	22	16	66.1	82.9	24	8.69
4	88	21	0	1	26.27	22.75	24.51	19.47	14.9	20	16	58.1	90	33	9.54
4	89	22	0	0	25.05	25.14	25.095	12.94	20.1	15.6	13.7	55.6	84.4	34.4	14.9
4	90	25	0	1	25.65	25.07	25.36	16.34	15	21.3	14.2	68.4	97.1	22.2	12.1

* L: the distance between sphere origins
H the height (H) of the plane S_H
L_L: the position of the lateral fitting plane
L_M: the position of the medial fitting plane
alpha_L: the angle between the horizontal plane and the lateral fitting plane
alpha_M: the angle between the horizontal plane and the medial fitting plane
beta_L: the angle between the sagittal plane and the lateral fitting plane
beta_M: the angle between the saggital plane and the medial fitting plane
